# Capillary telangiectatic osteosarcoma misdiagnosed as aneurysmal bone cyst in a 12-year-old girl: A case report

**DOI:** 10.1016/j.ijscr.2025.111174

**Published:** 2025-03-18

**Authors:** Xianyong Luo, Xinrang Chen

**Affiliations:** Department of Pediatric Surgery, The First Affiliated Hospital of Zhengzhou University, 450052 Zhengzhou City, Henan Province, China

**Keywords:** Telangiectatic osteosarcoma, Aneurysmal bone cyst, Pathological diagnosis, Chemotherapy

## Abstract

**Introduction and importance:**

Telangiectatic osteosarcoma (TOS) is a rare and aggressive subtype of osteosarcoma that is often misdiagnosed as more benign lesions, such as aneurysmal bone cysts (ABCs) or giant cell tumors (GCTs). The accurate differentiation between these conditions is crucial to ensure timely and appropriate treatment, as misdiagnosis can lead to delayed management and poor prognoses.

**Case presentation:**

We present the case of a 12-year-old girl who initially presented with left hip and proximal thigh pain, with imaging studies suggesting an ABC. Following curettage and grafting, the initial pathology report confirmed the diagnosis of ABC. However, the patient experienced rapid recurrence within two months, leading to further surgical intervention. A thorough re-evaluation of the pathological specimen revealed characteristics consistent with TOS, confirmed by immunohistochemical staining and molecular tests. The patient received four cycles of chemotherapy after the second surgery and underwent limb - salvage surgery 3 months after the operation.

**Clinical discussion:**

This case highlights the challenges in diagnosing TOS due to its histological similarity to other giant cell-rich lesions. The initial misdiagnosis delayed the commencement of appropriate treatment. However, upon correct identification, the patient was treated with an effective chemotherapy regimen, including cisplatin, doxorubicin, and ifosfamide, resulting in significant tumor regression and control of lung metastases.

**Conclusion:**

This case underscores the necessity for clinicians to maintain a high index of suspicion for TOS when evaluating aggressive bone lesions, particularly in pediatric patients. The importance of comprehensive pathological assessment, including immunohistochemistry and molecular analysis, cannot be overstated, as early and accurate diagnosis significantly influences treatment outcomes and prognosis.

## Highlights

• Rare TOS misdiagnosed as ABC in girl, stressing accurate giant cell assessment.
• Pathology/molecular analysis confirmed TOS post-ABC, stressing advanced diagnostics.
• Delayed TOS diagnosis; chemotherapy effective, improving prognosis.
• Limb-sparing surgery post-chemo successful in TOS despite prior misdiagnosis.
• High clinical suspicion for TOS needed in aggressive bone lesions for early treatment.

## 1 Introduction

Telangiectatic osteosarcoma (TOS) is a rare subtype of osteosarcoma, accounting for 2 % to 12 % of all osteosarcoma cases, with a predilection for the distal femur and proximal tibia [1], characterized by cystic spaces filled with blood and extensive necrosis, which can be difficult to differentiate from other lesions such as aneurysmal bone cyst (ABC) and giant cell tumor (GCT). Although TOS is uncommon, it is highly aggressive and prone to early metastasis, especially to the lungs. Accurate diagnosis is crucial for effective treatment, as TOS is highly responsive to chemotherapy. This case highlights the challenges in diagnosing TOS and emphasizes the importance of pathological and molecular analysis, as well as the role of chemotherapy in preventing poor outcomes due to delayed treatment.

## 2 Case presentation

A 12-year-old girl presented with a one-month history of left hip and proximal thigh pain. Initial X-ray and MRI revealed a cystic lesion in the proximal femur (Fig. 1A, Fig. 1B), which was suspected to be an aneurysmal bone cyst. The patient underwent curettage, bone grafting, and fixation with a proximal femoral PHP anatomical plate. Postoperative pathology revealed a giant cell-rich lesion, supporting the diagnosis of ABC.

**Fig. 1A:**
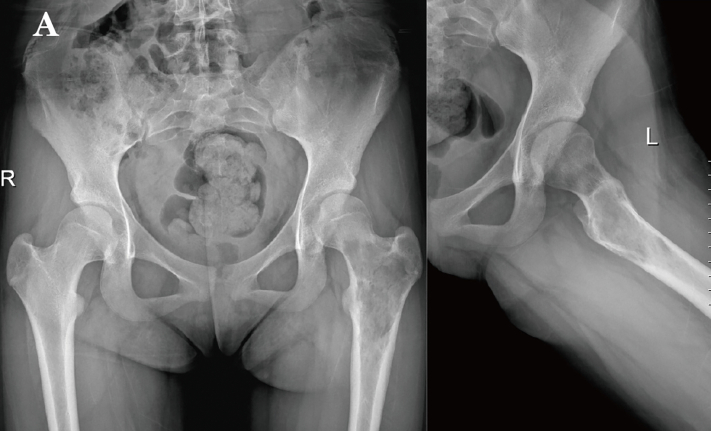
Preoperative X-ray revealed a cystic lesion in the proximal left femur.

**Fig. 1B:**
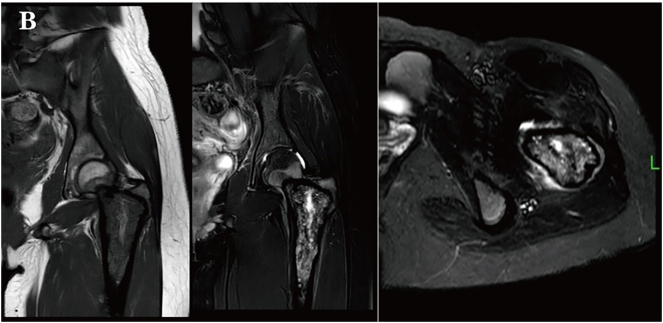
MRI demonstrated a mixed long T1 and long T2 signal in the proximal left femur.

Two months after surgery, without any external trauma triggers, the patient experienced a recurrence of pain in the left hip and developed movement disorders, unable to stand or walk, and follow-up imaging indicated progression of the lesion and hardware failure (Fig. 1C, Fig. 1D). Repeat surgery was performed, during **the 1.5-hour** operation, the steel plate and screws were removed. *Notably*, during the surgery, it was found that the intertrochanteric bone was destroyed and absorbed by the tumor. A large amount of blood clot-like tissue and loose bone particles were cleared. The remaining bone cortex was preserved, and a 3.5 mm Kirschner wire was inserted into the distal femur for skeletal traction. Pathological examination with immunohistochemical staining revealed SATB2 (+), CD68 (+), and Ki67 (+ at approximately 70 %). The lesion contained numerous giant cells with areas of necrosis, numerous mitoses, and focal osteoid formation, raising suspicion for telangiectatic osteosarcoma. Fluorescence in situ hybridization (FISH) molecular testing showed USP6 (−), H3F3A (−), and H3F3B (−), ruling out ABC and GCT. These findings supported the diagnosis of TOS. PET-CT revealed hypermetabolic activity at the femoral lesion, along with multiple small lung nodules and hypermetabolic left iliac lymph nodes (Fig. 1E), suggesting metastasis.

**Fig. 1C:**
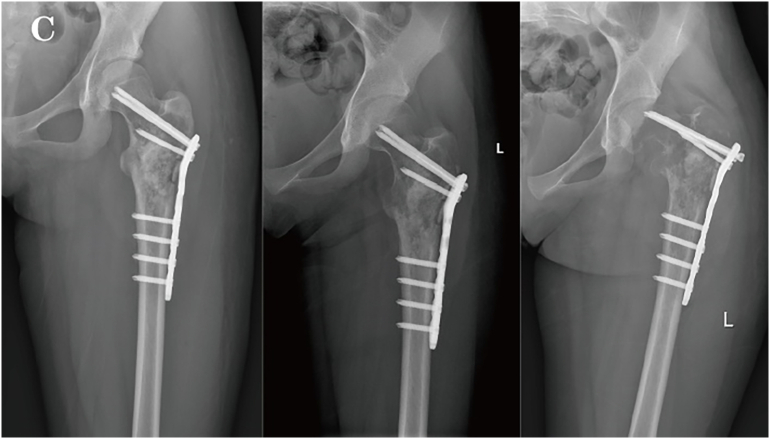
X-ray at two months postoperatively showed hardware displacement and aggressive bone changes.

**Fig. 1D:**
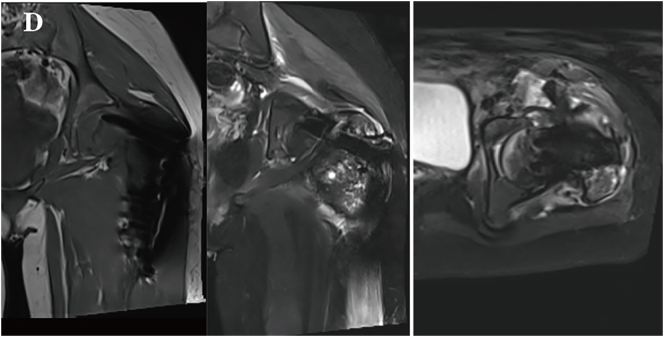
MRI showed cortical expansion with resorption and the presence of a soft tissue mass.

**Fig. 1E:**
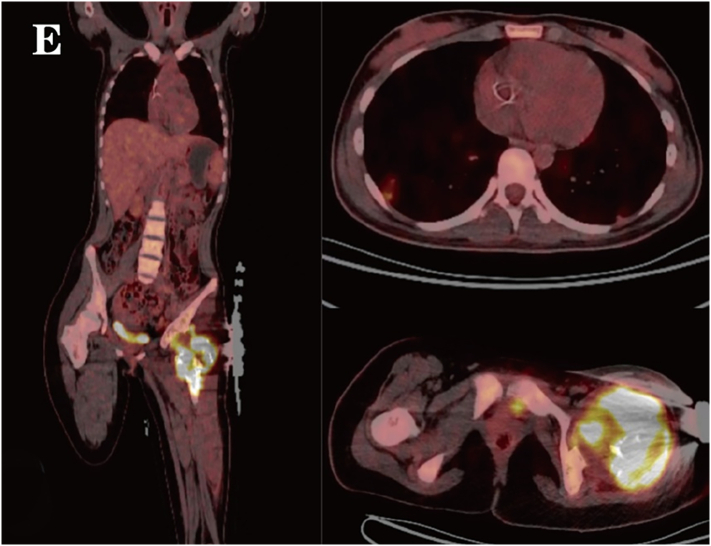
PET-CT revealed hypermetabolic activity in the soft tissue mass at the proximal left femur with cortical destruction, suggesting tumor invasion. Multiple hypermetabolic nodules in both lungs indicated metastasis.

The patient subsequently underwent chemotherapy with a regimen of cisplatin, doxorubicin, and ifosfamide for four times, with an interval of two weeks between each session. Each chemotherapy session lasted from 2 to 5 days, which led to significant reduction in the primary lesion and lung metastases (Fig. 1F). After the chemotherapy, the patient underwent limb-sparing surgery to remove the residual tumor (Fig. 1G). She tolerated the treatment well, and no disease progression was observed during the subsequent one-year follow-up. During follow-up, the patient exhibited *gait abnormalities* with *restricted hip and knee flexion* range of motion. Long-distance walking continued to require assistive devices such as crutches or wheelchairs. No complications including fever, bone pain, or wound infection were noted.

**Fig. 1F:**
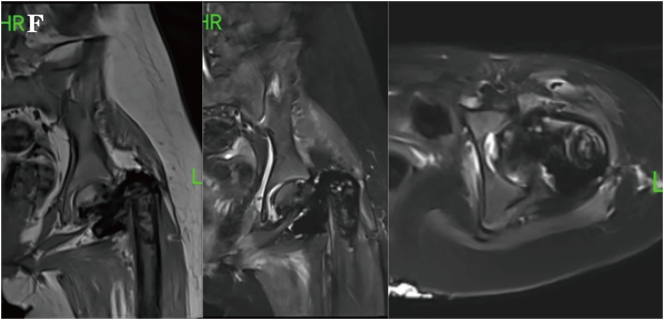
MRI after three cycles of chemotherapy showed a reduction in the size of the mass.

**Fig. 1G:**
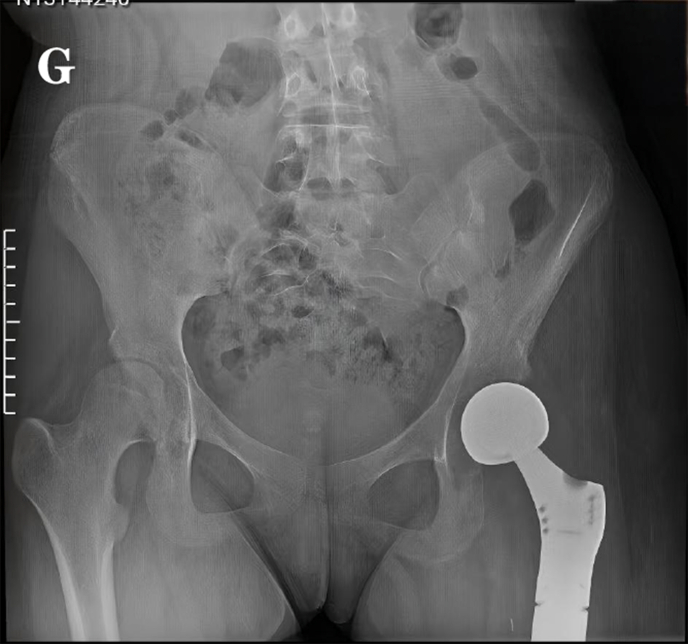
The X-ray image two months after the limb-sparing surgery.

## 3 Clinical discussion

Telangiectatic osteosarcoma (TOS) is a rare subtype of osteosarcoma, and frequently misdiagnosed as other benign cystic bone lesions such as aneurysmal bone cyst (ABC) due to their similar imaging features. Both TOS and ABC can present as cystic spaces with fluid-fluid levels on imaging; however, ABC typically demonstrates relatively mild bone destruction, characterized by expansile, lytic lesions with intact cortices and minimal periosteal reaction, without soft tissue involvement. In contrast, TOS exhibits more aggressive bone destruction with cortical disruption, periosteal reactions, and soft tissue masses, and MRI usually shows fewer fluid-fluid levels [2]. The presence of giant cells in TOS further complicates differentiation from giant cell tumor (GCT), which also exhibits aggressive features such as cortical expansion or destruction, and may display fluid-fluid levels. GCT is typically a lytic lesion with sharp, non-sclerotic borders and an eccentric location, often extending to the joint surface [3].

When imaging is inconclusive, pathological diagnosis becomes critical. Microscopically, TOS is characterized by hemorrhage and necrosis, with atypical stromal cells and occasional osteoid formation, while ABC and GCT lack malignant stromal cells. Molecular testing can further clarify the differential diagnosis: Osteosarcoma is usually associated with mutations in the retinoblastoma (RB) or TP53 genes. Both are tumor suppressor genes involved in cell cycle regulation. RB exerts its tumor - suppressing effect by regulating the progression of the cell cycle from the G1 to the S phase; TP53 is involved in DNA repair and the regulation of apoptosis. However, no specific diagnostic cytogenetic or molecular markers for TOS have been identified so far [4]. GCT frequently shows H3F3A or H3F3B mutations, while ABC is associated with USP6 gene rearrangements [5]. This case emphasizes the importance of pathological and immunohistochemical analysis, with molecular testing often playing a decisive role in the diagnosis (Table 1**)**.

**Table 1:** Comparative table for TOS, ABC and GCT differential diagnosis.

	TOS	ABC	GCT
Clinical	Aggressive, often misdiagnosed	Benign, non-aggressive	Locally aggressive
Radiology	Aggressive destruction, soft-tissue mass, fewer fluid levels	Expansile lytic lesion, intact cortex, fluid levels	Eccentric lytic lesion, joint involvement
Histopathology	Atypical stromal cells, osteoid	Blood-filled cysts, reactive stroma	Giant cells, mononuclear cells
Molecular	RB/TP53 mutations	USP6 rearrangements	H3F3A/B mutations

Despite its aggressive nature, TOS is highly sensitive to chemotherapy, likely due to its rich vascular structure, which facilitates better drug penetration into the tumor [6]. Studies have shown that the prognosis of TOS is similar to other high-grade osteosarcoma subtypes, with a 5-year overall survival rate of 66.8 %. The introduction of at least three active chemotherapy agents improves prognosis, and even in cases of pathological fractures, limb-sparing surgery is feasible due to the tumor's chemosensitivity [7]. Additionally, factors such as tumor volume and LDH levels are prognostic indicators, while pathological fractures and initial misdiagnosis do not significantly impact outcomes [8]. Another study suggests that Tumor size, location (in upper or lower extremities), age, gender, race, and socioeconomic status have minimal impact. However, distant metastasis significantly worsens prognosis. In terms of surgery, limb - salvage surgery is more favorable than amputation, while regional lymph node dissection is associated with poor outcomes. Chemotherapy is beneficial for survival, yet neoadjuvant chemotherapy shows no marked advantage over traditional chemotherapy [9]. In this case, the delay in appropriate treatment due to initial misdiagnosis did not negatively affect the patient's prognosis, as the tumor responded well to chemotherapy, allowing for limb-sparing surgery and control of lung metastases.

This case underscores the importance of considering TOS in the differential diagnosis of giant cell-rich lesions, particularly in cases of rapid recurrence or aggressive behavior. Early diagnosis and treatment are critical for optimizing patient outcomes, as timely chemotherapy can significantly reduce tumor burden and improve prognosis.

This study has been reported in line with the SCARE 2023 criteria [10]. The generalizability of this study is constrained due to the inherent limitations of single-case design in sample representativeness, potentially restricting the extrapolation of conclusions to wider demographic groups.

## 4 Conclusion

Telangiectatic osteosarcoma is a rare but highly aggressive malignancy that is often misdiagnosed as aneurysmal bone cyst or giant cell tumor. Accurate pathological evaluation is essential for diagnosis, and chemotherapy plays a key role in treatment. Conducting tests for new biological targets and developing multimodal therapies have emerged as the future research directions. This case demonstrates that even in instances of initial misdiagnosis, appropriate intervention can lead to a favorable prognosis, although the patient may endure additional physical and emotional burdens. Early recognition of TOS is crucial to avoid treatment delays and improve patient outcomes.

## CRediT authorship contribution statement

Data collection and analysis: Xianyong Luo and Xinrang Chen; drafting of the article: Xianyong Luo; crit ical revision of the article for important intellectual content: Xianyong Luo; study supervision: All the authors approved the final article.

## Informed consent

Written informed consent was obtained from the patient's parents/legal guardian for publication and any accompanying images. A copy of the written consent is available for review by the Editor-in-Chief of this journal on request.

## Ethical approval

This is a retrospective case report, and the Ethics Committee waived the ethical approval.

## Guarantor

Xianyong Luo.

The First Affiliated Hospital of Zhengzhou University.

## Research registration number

Not applicable.

## Funding

This study was not funded by any external sources.

## Declaration of competing interest

The authors declare no conflicts of interest.

## Data Availability

All data that support the findings of this study are included in this manuscript and its supplementary information files.
